# High-dose hook effect in prolactin macroadenomas: A diagnostic concern

**DOI:** 10.4103/0974-1208.74164

**Published:** 2010

**Authors:** Manika Agarwal, Ananya Das, A Santa Singh

**Affiliations:** Department of Obstetrics & Gynaecology, North Eastern Indira Gandhi Regional Institute of Health & Medical Sciences (NEIGRIHMS), Mawdiangdiang, Shillong, Meghalaya, India

Sir,

I have gone through the letter to the editor regarding laboratory concern for the high-dose hook effect in prolactin assays. The intensity of an antigen–antibody interaction depends primarily on the relative proportion of the antigen and the antibody. A relative excess of either will impair adequate immune complex formation. This is called the “high-dose hook effect” or the “prozone phenomenon.” Extremely high levels of prolactin (PRL) can interfere with the assay and produce low readings. This high-dose hook effect may occur because there is not enough antibody to bind to both ends of all antigenic (prolactin) peptides. Most of the PRL is now complexed to a single antibody. Only the few remaining PRL peptides are “sandwiched” and therefore detectable. This results in a falsely low PRL value. Hence, as the antigen concentrations increase, there is a proportional increase in assay titers up to a certain level. Antigen concentrations above this threshold level would “hook” down the assay values resulting in very low measurements.[1,2] In addition, high-antigen titers can directly dissolve the antigen–antibody complex.[1] In order to avoid the high-dose hook effect, the serum PRL should be estimated in appropriate dilution in all patients with large pituitary tumors. The high-dose PRL hook effect is observed particularly in patients with very large tumors. The immunoradiometric PRL assay must be performed with serum dilution in order to overcome the high-dose PRL hook effect in all new patients with pituitary macroadenomas who may have a prolactinoma.[3] Other suggested remedies for the hook effect include the use of an excess antibody, a cumbersome two-step procedure, and the use of a computer to predict the head to dilute serum samples.[1] Though repeatedly demonstrated in other immunoassays, the high-dose hook effect has only occasionally been observed in chemiluminescence assay systems for PRL estimation.[1]

Whatever the author has cited with references is no doubt of laboratory concern in prolactin assays, but has little relevance to our case report. Our case is not a patient of pituitary prolactinoma with moderate to severe hyperprolactinemia, where the high-dose hook effect of prolactin is of more significance. Moreover, our laboratory uses chemiluminescence assays for prolactin estimation which rarely shows fallacies due to the high-dose effect.
